# Investigation of the antigenicity and protective efficacy of *Leishmania* promastigote membrane antigens in search of potential diagnostic and vaccine candidates against visceral leishmaniasis

**DOI:** 10.1186/s13071-020-04138-7

**Published:** 2020-05-30

**Authors:** Sarfaraz Ahmad Ejazi, Smriti Ghosh, Anirban Bhattacharyya, Mohd Kamran, Sonali Das, Sudipta Bhowmick, Mehebubar Rahaman, Rama Prosad Goswami, Nahid Ali

**Affiliations:** 1 Infectious Diseases and Immunology Division, Indian Institute of Chemical Biology, Kolkata, West Bengal India; 2 Present Address: Department of Botany, Serampore College, Hooghly, Serampore, West Bengal India; 3 Present Address: Dr. Kanailal Bhattacharyya College, Dharmatala, Ramrajatala, Santragachi, Howrah, West Bengal India; 4 Department of Tropical Medicine, School of Tropical Medicine, Kolkata, West Bengal India

**Keywords:** Biochemistry, Parasitology, Leishmaniasis, Immunology, Diagnosis, Vaccination, Th1/Th2, Cytokines

## Abstract

**Background:**

Visceral leishmaniasis (VL), is a parasitic disease that causes serious medical consequences if treatment is delayed. Despite a decline in the number of VL cases in the Indian subcontinent, the commencement of the disease in newer areas continues to be a major concern. Although serological diagnosis mainly by immunochromatographic tests has been found to be effective, a test of cure in different phases of treatment is still desired. Even though a good prophylactic response has been obtained in murine models by a number of vaccine candidates, few have been proposed for human use.

**Methods:**

In this study, nine antigenic components (31, 34, 36, 45, 51, 63, 72, 91 and 97 kDa) of *Leishmania* promastigote membrane antigens (LAg), were electroeluted and evaluated through ELISA to diagnose and distinguish active VL from one month cured and six months post-treatment patients. Further, to investigate the immunogenicity of electroeluted proteins, human PBMCs of cured VL patients were stimulated with 31, 34, 51, 63, 72 and 91 kDa proteins.

**Results:**

We found that 34 and 51 kDa proteins show 100% sensitivity and specificity with healthy controls and other diseases. After six months post-treatment, antibodies to 72 and 91 kDa antigens show a significant decline to almost normal levels. This suggests that 34 and 51 kDa proteins are efficient in diagnosis, whereas 72 and 91 kDa proteins may be used to monitor treatment outcome. In another assay, 51 and 63 kDa proteins demonstrated maximum ability to upregulate IFN-γ and IL-12 with minimum induction of IL-10 and TGF-β. The results indicating that 51 and 63 kDa proteins could be strong candidates for human immunization against VL. In contrast, 34 and 91 kDa proteins demonstrated a reverse profile and may not be a good vaccine candidate.

**Conclusions:**

The preliminary data obtained in this study proposes the potential of some of the antigens in *Leishmania* diagnosis and for test of cure. Additionally, some antigens demonstrated good immunoprophylactic cytokine production through T cell-mediated immune response, suggesting future vaccine candidates for VL. However, further studies are necessary to explore these antigens in diagnosis and to access the long-term immune response.
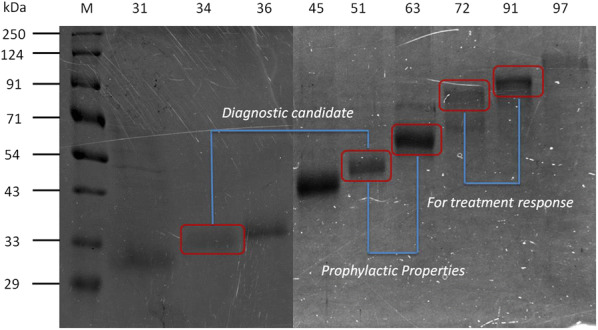

## Background

Despite a reduction in the number of visceral leishmaniasis (VL) cases in previously endemic areas, many regions still show the presence of disease burden and also spread into newer areas has been reported. Early diagnosis with complete treatment of cases, together with the development of a vaccine, should be considered as important solutions [1–3]. Clinical diagnosis of VL depends on the demonstration of parasites in spleen or bone marrow aspirates, a complicated procedure to perform, but still considered as the gold standard in routine diagnosis [4]. High levels of anti-leishmanial antibodies are developed during the acute disease, which is maintained up to several years and can be detected by various serological tests such as ELISA, IFAT and direct agglutination test (DAT). Development of the rK39 antigen-based strip test has brought a major advancement in VL diagnosis for practice in field settings. Nevertheless, the poor sensitivity of the rK39 in East Africa, and suboptimal performance in Brazil, necessitate the scope of research for better diagnostic tools [5]. Failure to differentiate between active and past infection is another limitation with the rK39 test. Since a single antigen is not effective for all endemic areas, newer antigens have been screened and evaluated for the serodiagnosis of VL. Recombinant leishmanial antigens such as rKLO8, rKE16 and rK28 are few of them [6–9]. In our previous studies, we have reported the diagnostic value of *Leishmania* promastigote membrane antigens (LAg) through various immunological techniques such as ELISA, immunoblot and dipstick test [5, 10, 11]. Moreover, anti-leishmanial antibodies in the sera of active and cured VL patients have shown variable reactivity against several proteins of LAg in the immunoblot assay [12].

*Leshmania donovani* infections in humans do not always result in disease manifestations. In VL-endemic areas self-resolving infection has also been observed by developing parasite-specific antibodies and/or T cell response [13, 14]. Furthermore, patients who have recovered from kala-azar are usually immune to reinfection, which suggests that vaccination against leishmaniasis should be feasible [15, 16]. Studies from animal models have shown that protection against *Leishmania* can be achieved using parasite-specific proteins, DNA or genetically attenuated parasites [17, 18]. Advances in our understanding of *Leishmania*-host interactions, *Leishmania* pathogenesis, protective immunity and the availability of the complete *L. donovani* genome sequence, could take this a step further. Reports from earlier studies in our laboratory have demonstrated the immunogenicity of *L. donovani* promastigote membrane antigens, either free or in liposomal preparations [19, 20]. In addition to inducing very good protection in the murine model, it could induce remarkable lymphoproliferation and protective cytokines (IFN-γ and IL-12) production in successfully treated kala-azar patients [21]. Similar results were also observed with soluble leishmanial antigens (SLA), partially purified from leishmanial membrane antigens, which when entrapped in cationic liposomes conferred almost complete protection as a prophylactic or therapeutic vaccine against *L. donovani* in BALB/c mice [22]. These data indicate that some of these peptides are more immunogenic than the others in experimental mouse models, and could be interesting to investigate their immunogenicity in humans.

Screening of the most immunodominant antigens of formerly tested purified antigens in response to the human immune system is an important task. On the other hand, differentiation between active and past infection is one of the major challenges for the serodiagnosis of VL. Moreover, antigens used in recent years are mostly recombinants that evade post-translational modification unlike purified antigens [23]. Therefore, in this study, we have evaluated purified leishmanial antigens, LAg and SLA, and electroeluted different fractions of LAg such as 31, 34, 36, 45, 51, 63, 72, 91 and 97 kDa proteins in their native state to differentiate active VL from healthy controls and cured individuals, through ELISA. We have also characterized 31, 34, 51, 63, 72 and 91 kDa proteins of LAg for immune stimulatory efficacy of PBMCs of cured VL patients through cytokine analysis as potential vaccine candidates.

## Methods

### Sample collection

Serum samples used in this study were collected from the School of Tropical Medicine (STM), Kolkata. Twenty-three VL patients were enrolled for the longitudinal study having single dose liposomal amphotericin B therapy (10 mg/kg). Blood samples were collected before the treatment, from active VL cases (AVL), one month after the treatment, from cured VL cases (CVL), and from follow-ups (FU) at approximately six months post-treatment. Sera was also collected from 23 other symptomatically similar diseases comprised of four samples each from malaria, tuberculosis, pneumonia, typhoid and viral fever, and one sample from a patient with a liver abscess, a patient with systemic lupus erythematosus, and a patient with pancreatitis. Sample collection continued with 23 healthy individuals as controls from the Indian Institute of Chemical Biology (IICB), Kolkata.

### Parasite culture and purification of leishmanial antigens

*Leishmania donovani* strain AG83 (ATCC^©^ PRA-413™) of the parasite was regularly maintained in hamsters. Amastigotes were isolated from sacrificed hamsters and allowed to transform into promastigotes in culture medium (M199) at 22 °C with supplements such as 10% heat-inactivated fetal bovine serum, 100 U/ml penicillin G sodium and 100 g/ml streptomycin sulfate. Promastigotes were subcultured through fresh medium passages and 3rd to 5th passage cultures were harvested and centrifuged to obtain cell pellets. Cell pellets were then washed in PBS and stored at − 20 °C until use.

*Leishmania* promastigote antigens (LAg) were purified from the cell pellet. In a typical experiment, cells were suspended in 5 mM Tris-HCl (pH 7.4) and vortexed for 12 min (2 min, 6 times) for the parasite membrane to become leaky. Parasites were then centrifuged (2310× *g*, 10 min, 4 °C) to collect the ghost membrane pellet which was then resuspended in the same buffer and subjected to ultrasonication (30 s, 6 times). The suspension was centrifuged (5190× *g*, 30 min, 4 °C) to obtain the antigens in the supernatant. The concentration of LAg was estimated by Lowry’s methods and stored at − 80 °C until further use. Soluble leishmanial antigen, SLA, was also purified from *Leishmania* promastigote culture similar to LAg with some modifications. The cell pellet was suspended in 1 mM EDTA, 5 µg leupeptin, 1 mM iodoacetamide and 1 mM phenylmethylsulfonyl fluoride in 5 mM Tris-HCl buffer, pH 7.4. The suspension was vortexed and centrifugation and sonication was performed as previously for LAg, followed by solubilisation in (1% w/v) octyl-β-d-glucopyranoside at 4 °C overnight. The next day the solubilised suspension was centrifuged at 100,000× g for 1 h. The supernatant collected contains SLA which was dialyzed and finally stored at − 80 °C after the protein concentration was estimated using Lowry’s method.

### SDS-PAGE and electroelution

Proteins were first denatured by the reducing agent β-mercaptoethanol (βME), and resolved by 10% SDS-PAGE. Different molecular weight proteins were separated from LAg (10 μg/lane) and SLA (5 μg/lane). The pattern of LAg and SLA proteins were visualized by Coomassie Blue. Rf values for the molecular weights of the respective proteins were determined by the automated Image Lab software (Bio-Rad, CA, USA) in comparison to the standards. For electroelution, 31, 34, 36, 45, 51, 63, 72, 91 and 97 kDa protein bands were excised from the LAg gel and subjected to electroelution as per the manufacturer’s protocol (Model-422; Bio-Rad). Subsequently, each protein was dialyzed against PBS. Electroeluted proteins after quantification were resolved on SDS-PAGE separately for reconfirmation of their molecular weights.

### Indirect ELISA for antibody detection

Indirect ELISA to capture antibodies was performed on 96-well flat bottom plates (Maxisorp Nunc; Thermo Fisher Scientific, MA, USA). In brief, wells were coated with 1 μg/well concentration of purified and electroeluted proteins with phosphate buffer (100 μl/well) and incubated overnight in 4 °C. The next day, antigen-coated wells were blocked with 1% BSA in PBS (200 μl/well) for 2 h at 37 °C. Subsequently, serum samples (1:2000) followed by peroxide conjugated antihuman IgG (1:4000) were applied to the wells in PBS buffer (100 μl/well) and incubated for 1 h at 37 °C. Plates were washed in each step with PBS and Tween 20 to remove any non-specific binding. Finally, wells were incubated in the substrate, o-phenylenediamine dihydrochloride (OPD), and H_2_O_2_ in the phosphate-citrate buffer (50 μl/well). The biological reaction was stopped with sulfuric acid and optical density values were acquired by using an ELISA plate reader (RS232C; Thermo Fisher Scientific, MA, USA) at a wavelength of 492 nm.

### Stimulation of PBMCs and cytokines analysis

Peripheral blood mononuclear cells (PBMCs) from heparinized blood samples of one month cured VL patients and healthy individuals were isolated by density gradient centrifugation using Histopaque-1077 (Sigma-Aldrich, MO, USA) and finally resuspended in RPMI 1640 medium with serum supplements and antibiotics. PBMCs obtained from each individual were cultured in triplicate (1 × 10^6^ cells/well) with and without antigen stimulation. Stimulation of cured VL and healthy PBMCs with LAg (12.5 µg/ml) and cured VL PBMCs with electroeluted antigens (1.5 µg/ml) 31, 34, 51, 63, 72 and 91 kDa, were performed at 37 °C in a CO_2_ incubator. Supernatant from the cultures were collected after 96 h and stored at − 20 °C for cytokine analysis. The level of cytokines, IFN-γ, IL-12, IL-10 and TGF-β, along with IFN-γ/ IL-10 and IFN-γ/ TGF-β, were measured by ELISA according to the manufacturers’ instructions (BD OptEIA ELISA kit; BD Biosciences, NJ, USA). Briefly, capture antibodies specific to the cytokines were coated in the wells of 96-well plates in carbonate buffer overnight at 4 °C. Subsequently, wells were blocked and incubated with culture supernatants for 1 h. Cytokine specific detection antibodies were used in the wells followed by TMB substrate. Optical density values were obtained in the ELISA reader at 450 nm.

### Mass spectrometry

Protein bands of molecular masses, 34 and 45 kDa were stained with Coomassie Blue and excised from 10% SDS-PAGE of LAg. The proteins imbibed in the gel bands were digested by an in-gel tryptic digestion kit, according to the protocol provided by the manufacturer (Thermo Fisher Scientific). Subsequently, co-crystallization of the digested peptides with the matrix was performed and subjected to MALDI-TOF/TOF for MS/MS spectra (Applied Biosystems, CA, USA). Data obtained from mass spectrometry were identified through the MASCOT search engine (http://www.matrixscience.com/search_form_select.html).

### Statistics

Statistical studies were conducted with Graph Pad Prism software version V. A two-tailed Student’s t-test was performed for paired and unpaired samples from indirect ELISA. *P*-values less than 0.05 were considered significant with 95% confidence intervals. Cut-off values were determined by the receiver-operator curve (ROC), where 100% sensitivity was obtained. The difference in cytokine production was determined by the two-tailed unpaired Student’s t-test.

## Results

### Immunoreactivity of LAg and SLA with *Leishmania* infected human serum samples

**Fig. 1:**
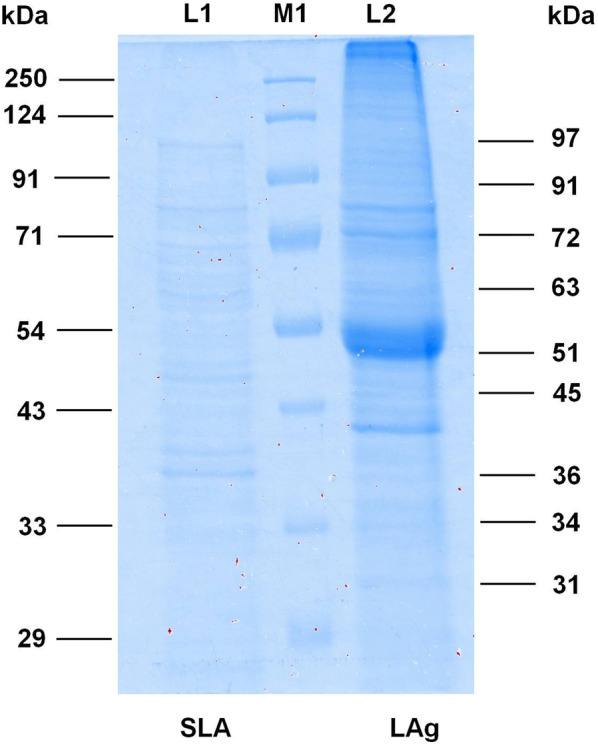
SDS-PAGE of purified proteins. Antigens, SLA and LAg were separated using SDS-PAGE and stained with Coomassie Blue to observe the individual proteins of each antigen. Lane M1: protein molecular weight markers; Lane L1: SLA; Lane L2: LAg

*Leishmania* promastigote membrane antigens (LAg) and soluble leishmanial antigens (SLA) were isolated from *L. donovani* promastigote culture and subjected to 10% polyacrylamide gel electrophoresis. LAg and SLA were resolved in their components of different molecular weights by SDS PAGE. LAg contains approximately 25–30 immunogenic proteins whereas SLA is a mixture of about ten dominant protein fractions (Fig. 1).

**Fig. 2:**
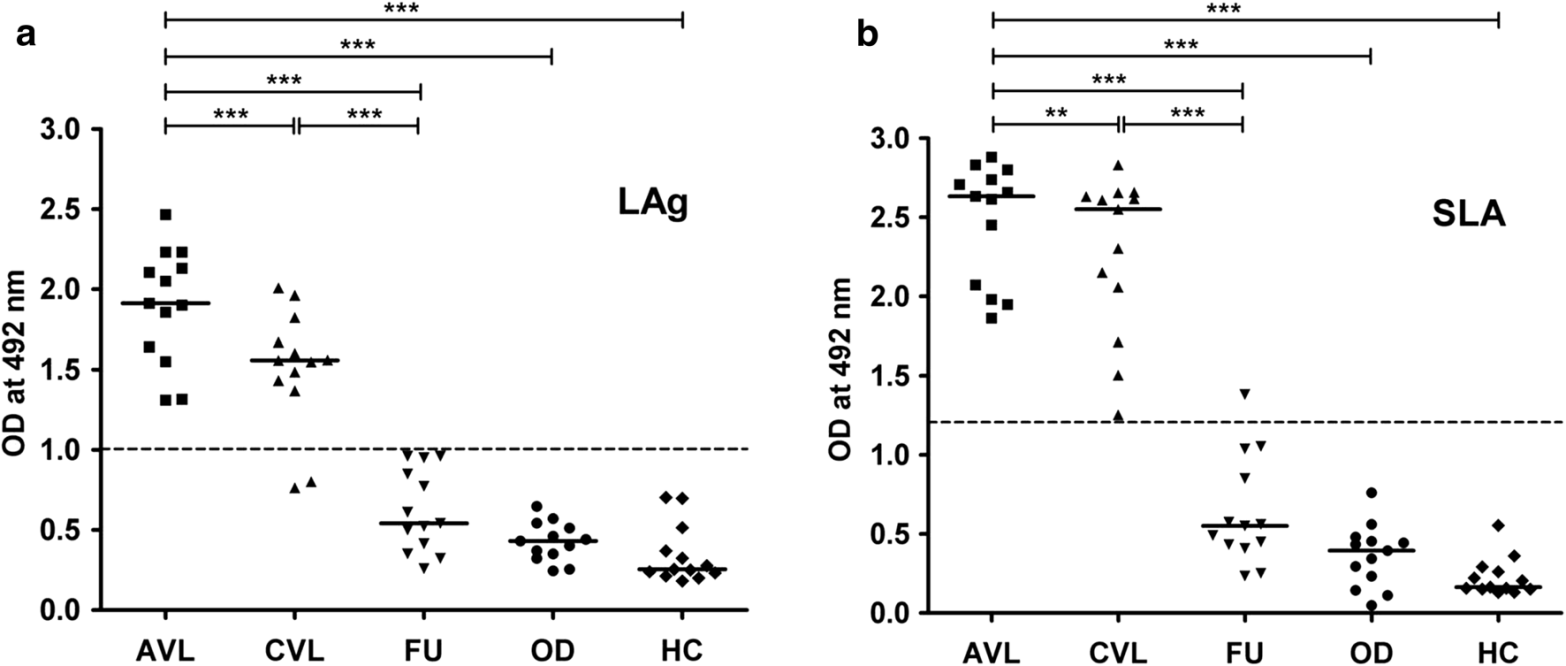
Indirect ELISA using purified antigens LAg and SLA. Optical density values were obtained by ELISA for the detection of serum antibodies against antigens LAg (**a**) and SLA (**b**). Paired serum samples (*n* = 13) from confirmed active VL cases before the treatment (AVL), cured VL one month after treatment (CVL), and six-month follow-ups (FU) were investigated against purified leishmanial antigens, LAg and SLA. Serum samples from 13 healthy individuals (HC) and 13 from other diseases were also tested. The cut-off values were selected from the ROC curve where 100% sensitivity and specificity were achieved. Each point represents an average of triplicate values obtained from a single sample

One of the most challenging objectives for *Leishmania*-specific antibody-based serodiagnosis is to differentiate an active VL patient from a patient with a past infection. In this regard reactivity of LAg and SLA with paired serum samples of 13 VL patients before treatment, one month cured and patients followed-up after six months, were evaluated by ELISA. We observed that there was a comparatively higher antibody titer in the active VL sera against LAg in comparison to other study groups. However, cured VL patients, just after treatment, showed a significant decrease in antibody titer but 84.16% positivity in ELISA (Fig. 2a). In the follow-up group, sera after six months of treatment showed a remarkable decrease in the antibody titer with no positive reactivity against LAg and the mean was below the cut-off line. No cross-reaction of LAg was found with 13 other diseases and 13 healthy controls tested. A similar assay was also performed with SLA, where both active and one month cured VL patients showed 100% positivity with SLA (Fig. 2b). However, 7.69% of the follow-up patients were still positive with SLA. Healthy controls and other diseases, however, did not show cross-reactivity with SLA. These results reveal that LAg and SLA have the strong serodiagnostic potential for VL. Both LAg and SLA could not satisfactorily differentiate active VL from past infections following one month post-therapy. However, after six months of infection, antibody titers significantly decreased below the cut-off and became100% negative for LAg. Therefore, in the next step, we isolated different components of LAg for immunoreactive studies of individual antigens.

### Electroelution of LAg antigens

**Fig. 3:**
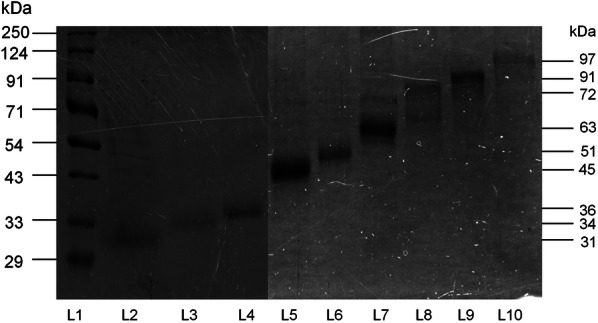
SDS-PAGE of electroeluted proteins of LAg. Electroeluted antigens 31, 34, 36, 45, 51, 63, 72, 91 and 97 kDa were run on 10% SDS-PAGE. Lane L1: marker; Lane L2: 31 kDa; Lane L3: 34 kDa; Lane L4: 36 kDa; Lane L5: 45 kDa; Lane L6: 51 kDa; Lane L7: 63 kDa; Lane L8: 72 kDa; Lane L9: 91 kDa; Lane L10: 97 kDa. The figure depicted here was obtained from two separate gels

To access the immunoreactive potential of different protein fractions of LAg, nine polypeptides of LAg, 31, 34, 36, 45, 51, 63, 72, 91 and 97 kDa were eluted out electrophoretically from Coomassie Blue-stained gels and subjected to SDS-PAGE (Fig. 3). To differentiate active disease from cured VL and follow-up patients, ELISA was carried out with all nine electroeluted antigens.

### Reactivity of electroeluted proteins in ELISA

Nine electroeluted antigens were evaluated in ELISA for their ability to distinguish the active disease from other diseases and healthy controls as well as from the cured VL and follow-up samples. Cut-off values were set for each eluted antigen where antibody titer above the cut-off line was considered as positive and below as negative. The cut-off values for VL positivity were obtained from the ROC curve, where maximum sensitivity and specificity were achieved in comparison with healthy controls. Out of the nine antigens, 100% sensitivity with active VL sera was observed with all electroeluted antigens. The specificity of the antigens was calculated based on the cross-reactivity of the antigens with the healthy controls and other diseases. With the healthy control samples tested, the mean optical density values for all the electroeluted antigens were below the cut-off line. 31, 34, 36, 51 and 97 kDa proteins were found to be 100% specific, whereas antigens 63 and 91 kDa showed more than 90% specificity. The reactivity of eluted antigens with other diseases showed 100% specificity with 34 and 51 kDa antigens, followed by 91 and 97 kDa with 95.75% specificity. Therefore, for the purpose of diagnosis, 34 and 51 kDa proteins were found to be the best to distinguish active VL from healthy controls and other diseases. The percent sensitivity and specificity of all the electroeluted antigens are listed in Table 1.

**Table 1:** Sensitivity and specificity of purified and electroeluted antigens with human sera

Purified and electroeluted antigens (kDa)	Sensitivity to active VL patients in % (*n*/*N*)	Sensitivity to 1-month treated VL patients in % (*n*/*N*)	Sensitivity to 6-month follow-up patients in % (*n*/*N*)	Specificity to other diseases in % (*n’*/*N*)	Specificity to healthy samples in % (*n’*/*N*)
LAg	100 (13/13)	84.16 (11/13)	0 (0/13)	100 (13/13)	100 (13/13)
SLA	100 (13/13)	100 (13/13)	7.69 (1/13)	100 (13/13)	100 (13/13)
31	100 (23/23)	60 (14/23)	26.08 (6/23)	39.1 (9/23)	100 (23/23)
34	100 (23/23)	73.91 (17/23)	13.04 (3/23)	100 (23/23)	100 (23/23)
36	100 (23/23)	69.56 (16/23)	78.26 (18/23)	40 (8/23)	100 (23/23)
45	100 (23/23)	100 (23/23)	60.86 (14/23)	30.43 (7/23)	60.86 (14/23)
51	100 (23/23)	91.30 (21/23)	30.43 (7/23)	100 (23/23)	100 (23/23)
63	100 (23/23)	47.82 (11/23)	4.34 (1/23)	91.30 (21/23)	95.65 (22/23)
72	100 (23/23)	39.1 (9/23)	0 (0/23)	65.21 (15/23)	73.91 (17/23)
91	100 (23/23)	8.69 (2/23)	0 (0/23)	95.75 (22/23)	91.30 (21/23)
97	100 (23/23)	78.23 (18/23)	86.95 (20/23)	95.75 (22/23)	100 (23/23)

*Abbreviations*: n, no. of positive samples in each group; N, total no. of samples tested in each group; n’, no. of negative samples in each group

**Fig. 4:**
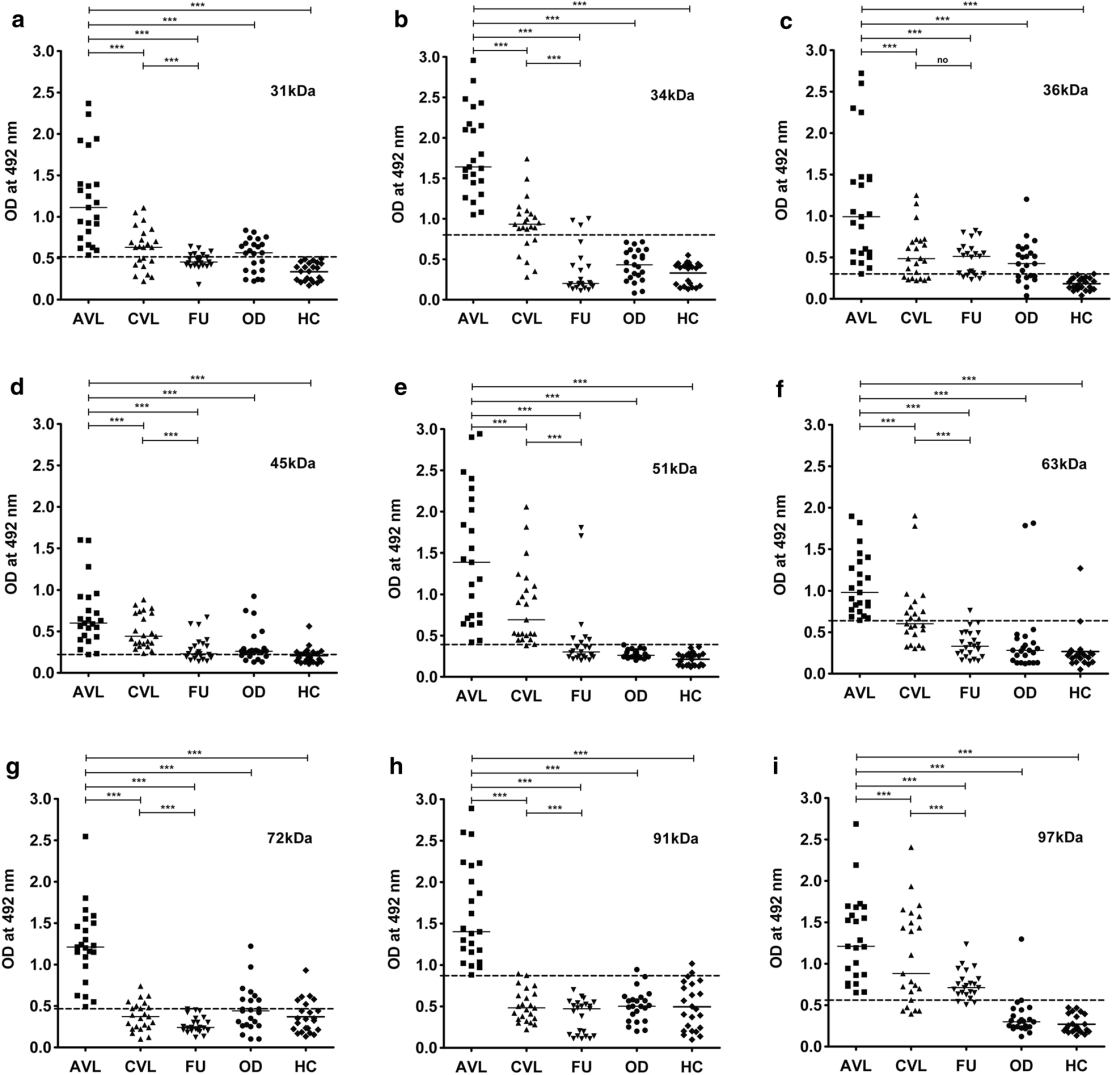
Indirect ELISA using electroeluted antigens. IgG reactivity of eluted antigens 31 (**a**), 34 (**b**), 36 (**c**), 45 (**d**), 51 (**e**), 63 (**f**), 72 (**g**), 91 (**h**) and 97 kDa (**i**) with serum samples from 23 VL patients at three different time points that is active VL before the treatment (AVL), after one month cured VL (CVL), and after six months follow-up cases (FU). The study also included sera from symptomatically similar other diseases (OD; *n* = 23) and healthy controls (HC; *n* = 23). The horizontal lines denote the mean value for each group. The dotted lines denote the cut-off values selected from the ROC curve where maximum sensitivities and specificities were achieved. Each point represents an average of triplicate values obtained from a single sample

The study to differentiate active VL from cured and past infections using electroeluted antigens demonstrated considerable differences among the patient groups (Fig. 4). There was a statistically considerable decline in serum IgG levels against all the antigens in the one month cured patient sera compared to their respective active disease sera. However, except for 63, 72 and 91 kDa, none of the antigens showed mean optical density values below its cut-off. Recognition of electroeluted antigens with sera after one month cure ranged from 8.69–100% which is still positive and not ideal for the differentiation of the diseased state from cure. Follow-up patients who recovered from VL six months post-treatment showed a significant decline in the IgG levels against 31, 34, 51, 63, 72, and 91 kDa antigens and their levels were comparable to the healthy control sera where the mean was below the cut-off. Amongst all the antigens, 72 and 91 kDa showed the most promising results, with 100% negative reactivity in follow-up patients.

### Evaluation of electroeluted proteins for cytokine analysis from human PBMCs

**Fig. 5:**
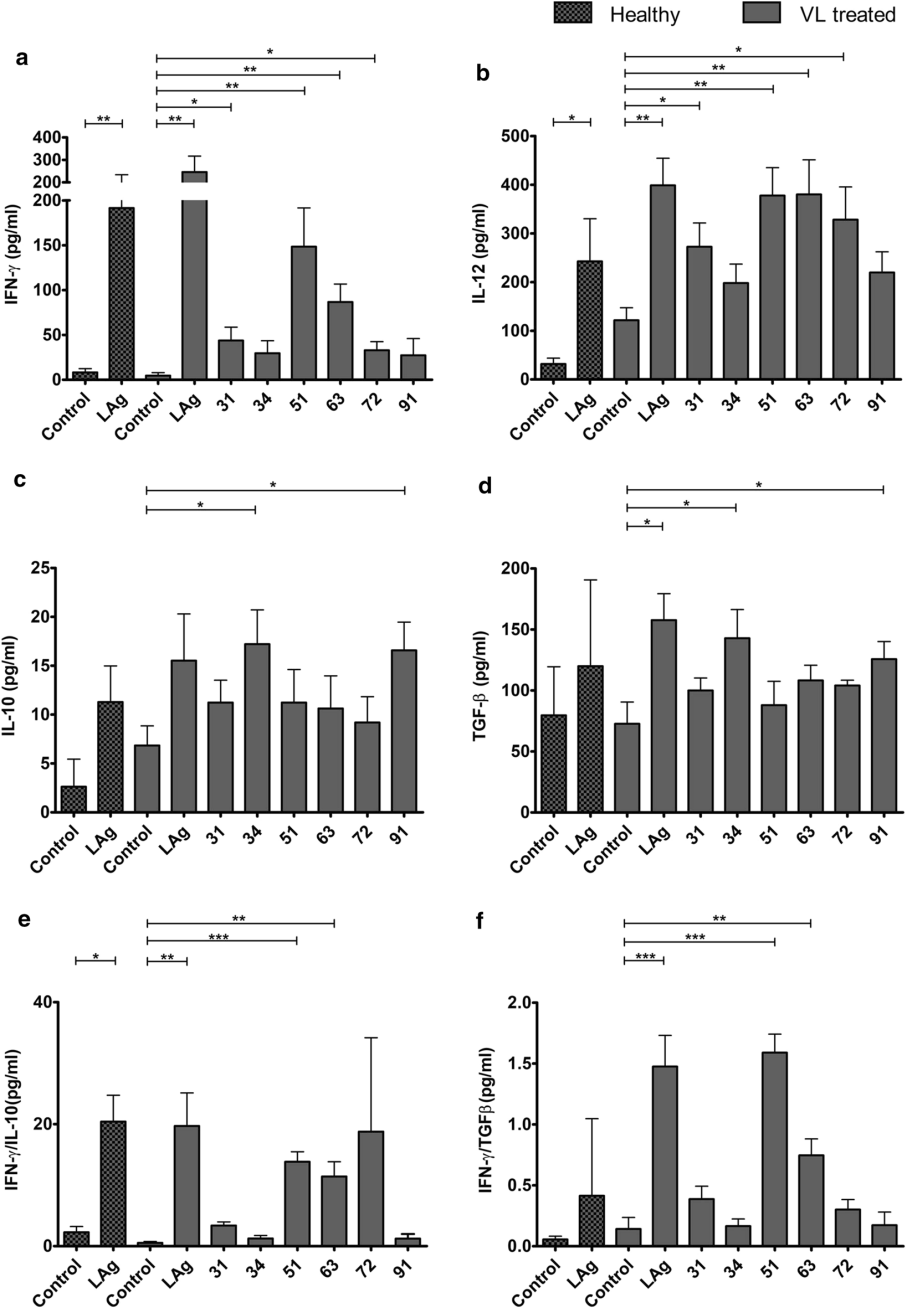
Sandwich ELISA for the cytokine assay. *In vitro* cytokine production is shown by the PBMCs of VL-treated and healthy individuals in ELISA upon stimulation with LAg and by the PBMCs of treated VL patients with electroeluted 31, 34, 51, 63, 72 and 91 kDa proteins for 96 h, (*n* = 6). Unstimulated PBMCs were included here as control. Cytokines were measured by ELISA for IFN-γ production (**a**), IL-12 production (**b**), IL-10 production (**c**), TGF-β production (**d**), IFN-γ/IL-10 ratio (**e**) and IFN-γ/ TGF-β ratio (**f**). Unpaired Studentʼs t-tests were used to analyse the data

Immunity to leishmaniasis is known to depend on protective cellular responses against the parasites. In order to assess the immunogenicity of LAg and eluted proteins, 31, 34, 51, 63, 72 and 91 kDa antigens were used to stimulate the PBMCs of cured VL patients for the production of protective cytokines IFN-γ and IL-12, and anti-inflammatory cytokines, IL-10 and TGF-β, which promote disease progression. IFN-γ is the most dominant Th1 cytokine required to control *Leishmania* infection. Analysis of cytokines revealed variable levels of IFN-γ production when PBMCs were stimulated with electroeluted antigens, whereas unstimulated cultures produced negligible IFN-γ (Fig. 5a). Prominent levels of IFN-γ were produced by the LAg-stimulated PBMCs of VL treated and healthy individuals as compared to unstimulated PBMCs. Significantly high levels of IFN-γ were produced by 51 and 63 kDa antigens followed by 31 and 72 kDa antigens when compared with unstimulated cultures. In comparison, 34 and 91 kDa antigens produced low levels of IFN-γ. IL-12 is another protective cytokine produced by the macrophages and in turn, activates the T cells to produce IFN-γ and accelerates the leishmanicidal function. Healthy and treated PBMCs exhibited significant IL-12 induction against LAg (Fig. 5b). Similar to the LAg stimulated cultures, significantly elevated levels of IL-12 were produced by 51and 63 kDa antigens followed by 31 and 72 kDa. 34 and 91 kDa antigens produced comparatively lower levels of IL-12.

IL-10 and TGF-β are two immunosuppressive cytokines that promote parasite survival and help in disease progression in VL infection. Our study demonstrated that LAg stimulated PBMCs from VL treated and healthy subjects produced moderate levels of IL-10. Among electroeluted antigens, 34 and 91 kDa antigens showed maximum levels of IL-10. In contrast, comparatively low levels of IL-10 were produced by 31, 51, 63 and 72 kDa electroeluted antigens (Fig. 5c). Low levels of TGF-β were produced by the healthy PBMCs than the cured PBMCs against LAg, whereas 31, 51, 63 and 72 kDa antigens produced median levels of TGF-β by the treated PBMCs (Fig. 5d). Again, 34 and 91 kDa antigens produced the highest levels of TGF-β similar to that produced by LAg stimulated cultures (Fig. 5d). Ratios of IFN-γ and IL-10 as well as IFN-γ and TGF-β were plotted in Fig. 5e, f.

### Identification of electroeluted proteins

Out of nine proteins of LAg evaluated in this study, except 34 and 45 kDa proteins, all proteins were identified in the earlier reports (Table 2). Therefore, we identified the remaining two proteins through MALDI-TOF mass spectrometry and matched with the *Leishmania* sequence. The 34 kDa protein was identified as *Leishmania* analogue of the receptors of activated C kinase (LACK) whereas the 45 kDa protein was β tubulin (Additional file 1: Figure S1, Additional file 1: Figure S2).

**Table 2:** List of electroeluted fractions of LAg and corresponding proteins

Electroeluted proteins (kDa)	Protein name	References
31	ATP synthase α chain	[29]
34	*Leishmania* analogue of the receptors of activated C kinase (LACK)	Present study
36	Elongation factor 1-α	[31]
45	β tubulin	Present study
51	β tubulin	[29]
63	Glycoprotein	[24]
72	Heat shock 70-related protein 1 mitochondrial precursor (HSP 70)	[29]
91	β tubulin	[29]
97	Not yet indentified from LAg/ may be nucleoporins-93 (NUP-93)	[35]

## Discussion

Visceral leishmaniasis is one of the foremost fatal infectious diseases for which no effective vaccine is currently available. Moreover, there is scope for finding better and effective tests for its diagnosis and to access treatment response. In this study, we have isolated and evaluated several *Leishmania* promastigote membrane antigens which can be used as probable candidates for diagnosis, test of cure as well as for vaccine development.

Estimating parasites microscopically from tissue aspirates is considered the gold standard for diagnosis but bears the risk of internal bleeding, pain, and dependence on a trained person is the major hurdle. VL is also characterized by the production of *Leishmania*-specific antibodies during disease. Hence in the last two decades, several serological methods have been evaluated largely using recombinant antigens with varying degrees of sensitivity and specificity. However, defined antigens do not undergo post-translational modifications such as glycosylation. Therefore, these antigens lack carbohydrate and lipid moieties as present in the native state of the antigen [23]. Thus, antigens in their native state were electroeluted to validate their diagnostic properties. Additionally, most of the serological tests are for the diagnosis of active VL conditions; therefore, assessing treatment efficacy remains an important challenge for VL management and elimination. In the present study, two purified leishmanial antigens, LAg and SLA, together with nine electroeluted proteins of LAg were evaluated for serodiagnosis of VL as well as to differentiate active VL from past infection. LAg has previously been reported to demonstrate a strong potential for the diagnosis of VL by detecting antigen-specific antibodies in serum and urine samples [10, 24]. SLA has also been reported in vaccine-mediated protection in murine VL [22, 25]. However, there is no study to investigate the different components of these purified antigens separately for diagnosis and to investigate their reactivity during different treatment phases. Herein, we observed that there is a significant decline in the *Leishmania*-specific antibody titers in the patients’ sera after one month of treatment for both LAg and SLA, However, they were still positive at the set cut-off values. Sera collected after six months of treatment demonstrated a sharp decline in the antibody titers against both LAg and SLA. Antibody levels for SLA remained above the cut-off line in some patients after six months follow-up in contrast to LAg, where at this time point, antibodies decreased to the level below the cut-off. Therefore, absolute differentiation between active VL sera from past infections is possible with LAg. In search of more defined immunoreactive antigens of LAg, nine antigens, 31, 34, 36, 45, 51, 63, 72, 91 and 97 kDa, were electroeluted to study their reactivity individually. We found that few electroeluted antigens introduced improvement over total antigen LAg, some of which hold a strong potential for serodiagnosis of VL. Antigens 31, 34, 36, 51 and 97 kDa were found to be 100% sensitive to detect active VL cases with 100% specificity against healthy controls. Antigens 34 and 51 kDa differentiated active VL and other diseases with 100% specificity. On the basis of the overall sensitivity and specificity of the antigens, these two antigens could be prospective candidates for future serodiagnosis of VL.

In kala-azar therapy, the initial cure is determined just after completion of treatment and a definite cure is determined six months post-treatment. Therefore, it is more important to distinguish the active disease from past infections of VL after at least six months of therapy. Very few studies have been undertaken to distinguish active VL from past infections of VL using serum samples. Antibody levels in sera were found to fall in response to rK26 and rK18 antigens after six months of treatment [26]. Studies of Indian and Sudanese *L. donovani* patient sera with crude antigen showed a significant decrease in IgG1 antibody levels after six months of cure [27]. A recent study with rK39 antigen demonstrated a greater decline in IgG1 isotypes in follow-up patients than IgG [28]. Therefore, to distinguish the active disease from past infections of VL, an antigen with high sensitivity for active VL may or may not be a good candidate for use in a test of cure. In our study, we were to some extent successful in differentiating active disease from past infections of VL using the electroeluted antigens. Interestingly, serum samples after six months of cure did not show reactivity with 72 and 91 kDa antigens. The other antigens which also hold a strong potential to distinguish active VL from past infections were 34 and 63 kDa proteins, and all these antigens had high reactivity with diseased VL sera and low reactivity with follow-up patient sera.

Most of the proteins electroeluted in this study have been identified through MALDI MS/MS in earlier reports [24, 29]. The role of these parasitic proteins in disease progression has been ascertained in mouse models from several previous studies [29–35]. The 31 kDa protein was identified as ATP synthase α chain of *Leishmania* [29]. ATP synthase complex subunits are functionally associated with the membrane and thus can be potential targets for drugs, diagnostic probes or vaccine components against *Leishmania*. A protein with a molecular weight of 34 kDa was identified in this study through mass spectrometry and recognized as a *Leishmania* analogue of the receptors of activated C kinase (LACK). LACK has been reported to induce a strong parasite-specific immune response and protects experimental mice against *L. donovani* infection [30]. The 36 kDa protein was identified as elongation factor 1-α (EF1-α), a translation factor that induces a cellular proliferative response and imparts long term immunity [31]. The 45 kDa protein was identified in the present study as beta-tubulin. The 51 kDa protein was also identified as beta-tubulin in our earlier reports, which exhibits high levels of protective cytokines and reduced infection in mice [29]. The 63 kDa protein is a membrane-anchored glycoprotein that corresponds to the infective stage and virulence of the parasite [32]. Another 72 kDa protein belongs to a set of highly evolutionary conserved heat-shock proteins. They behave as chaperones and induced Th1-type of cellular responses in cured patients and hamsters [33, 34]. The 91 kDa protein showed homology with β-tubulin but did not show immunogenicity and protective immunity as compared to the other tubulins in the experimental *L. donovani* vaccine study [29]. The 97 kDa protein was recently identified in both promastigote and amastigote forms of parasite as nucleoporins 93 (NUP93) and found to be an immunoprophylactic agent against *Leishmania* [35].

Identification of key antigenic targets of the protective human immune response against VL is central to the development of an efficacious vaccine against *Leishmania.* Cell-mediated immune responses are the key determinants for the natural course of *Leishmania* infection. Although human VL is known to elicit a mixed Th1 and Th2 response, protective immunity is achieved when the cell-mediated Th1 response is predominant. Thus, antigens that predominantly stimulate the Th1 response are considered as potential protective antigens [36]. The IL-12 driven IFN-γ dominated Th1 response is associated with resistance to infection against *Leishmania*, as it has a direct effect on the macrophage microbicidal response and other effector killing mechanisms [37]. Conversely, antigens that predominantly stimulate the Th2 response from these cells have been regarded as of lesser interest as vaccine candidates because they are likely to be associated with pathology [38]. IL-10 and TGF-β are the major immunosuppressive cytokines in VL and they can modulate macrophages and T cell functions and promote multiplication of *Leishmania* parasites as evident in patients with VL [21].

Studies have evaluated purified and recombinant proteins as well as whole parasite lysate for potential immunostimulatory activity in experimental models. Hence, it is important to evaluate leishmanial antigens for cellular immune responses in humans. Our earlier report reveals that entrapment of LAg in the cationic liposomes conferred significant levels of protection against infection in hamsters and BALB/c mice [39]. Immunodominant proteins of LAg, 31, 51, 63, 72 and 91 kDa antigens elicited a protective immune response in an experimental model [29]. Since PBMCs derived from VL-treated patients were exposed to *Leishmania* infection they showed an increase in protective cytokine response against LAg in the present study. However, PBMCs derived from healthy individuals also showed similar results suggesting the role of LAg antigens in prophylaxis. We characterized the immune response through cytokine analysis of six electroeluted antigens of LAg, 31, 34, 51, 63, 72 and 91 kDa that were recognized by sera of active and cured VL patients. Although the six antigens were able to stimulate PBMCs from cured VL individuals, the response was quite variable. The 51 and 63 kDa antigens induced maximum elevation of protective Th1 cytokines IFN-γ and IL-12, similar to that of LAg, followed by 31 and 72 kDa antigens that produced comparatively lower levels of IFN-γ and IL-12. Antigens 34 and 91 kDa, however, did not show significant production of IFN-γ and IL-12. Lower levels of immunosuppressive cytokines IL-10 and TGF-β were secreted by 31, 51, 63 and 72 kDa antigens. In contrast, 34 and 91 kDa antigens produced significantly higher levels of the suppressive cytokines, comparable to LAg. Therefore, these two antigens could be the reason for high IL-10 and TGF-β production from LAg. Moreover, the ratio of IFN-γ/IL-10 and IFN-γ/ TGF-β of treated patients also demonstrated the protective immune profile against LAg and 51 and 63 kDa among electroeluted antigens.

From this study, 51 and 63 kDa antigens may be considered to be the most potent vaccine candidates based on the cytokine analysis. The 31 and 72 kDa antigens were found to be less immunogenic and 34 and 91 kDa antigens did not show any potential to be considered as a vaccine candidate. Although the diagnostic and immunoprophylactic responses of antigens were clearly described in these preliminary experiments, the number of samples used was small. We therefore consider this as a limitation of our work and will try to overcome this in the next phase of the study.

## Conclusions

We report here the evaluation of electroeluted *Leishmania* membrane proteins some of which showed high sensitivity for active VL cases, as well as ability to differentiate from past VL infection. Thus they can be used as probable diagnostic candidates as well as for test of cure. Moreover, some of the electroeluted antigens stimulate significant protective cytokines IFN-γ and IL-12, and low levels of disease progressive cytokines, IL-10 and TGF-β, from human PBMCs after cure. Therefore, they may be considered as potential subunit vaccine candidates against VL.

## Supplementary information

**Additional file 1: Figure S1.** MALDI-TOF spectra of the tryptic fragments obtained from the peptide 34 kDa. **Figure S2.** MALDI-TOF spectra of the tryptic fragments obtained from the peptide 45 kDa.

## Data Availability

All data generated or analysed during this study are included in this published article and its additional file.

## Abbreviations

VL: visceral leishmaniasis
HIV: human immunodeficiency virus
LAg: *Leishmania* promastigote membrane antigens
SLA: soluble leishmanial antigen
ELISA: enzyme-linked immunosorbent assay
DAT: direct agglutination test
IFAT: immunofluorescence antibody test
MALDI-TOF: matrix assisted laser desorption/ionization-time of flight
SDS-PAGE: sodium dodecyl sulfate-polyacrylamide gel electrophoresis
EHC: endemic healthy control
NEHC: non-endemic healthy control
OD: other diseases
HC: healthy control
AVL: active visceral leishmaniasis
CVL: cured visceral leishmaniasis
FU: follow-up patients
