# Supplementary material for: Investigation of the antigenicity and protective efficacy of Leishmania promastigote membrane antigens in search of potential diagnostic and vaccine candidates against visceral leishmaniasis
Source: Parasit Vectors. 2020 May 30;13:272. doi: 10.1186/s13071-020-04138-7 (PMC7260476; doi:10.1186/s13071-020-04138-7)
Supplement: Supplementary file 1 — Additional file 1: Figure S1. MALDI-TOF spectra of the tryptic fragments obtained from the peptide 34 kDa. Figure S2. MALDI-TOF spectra of the tryptic fragments obtained from the peptide 45 kDa. [file 13071_2020_4138_MOESM1_ESM.pdf]

Additional file 1

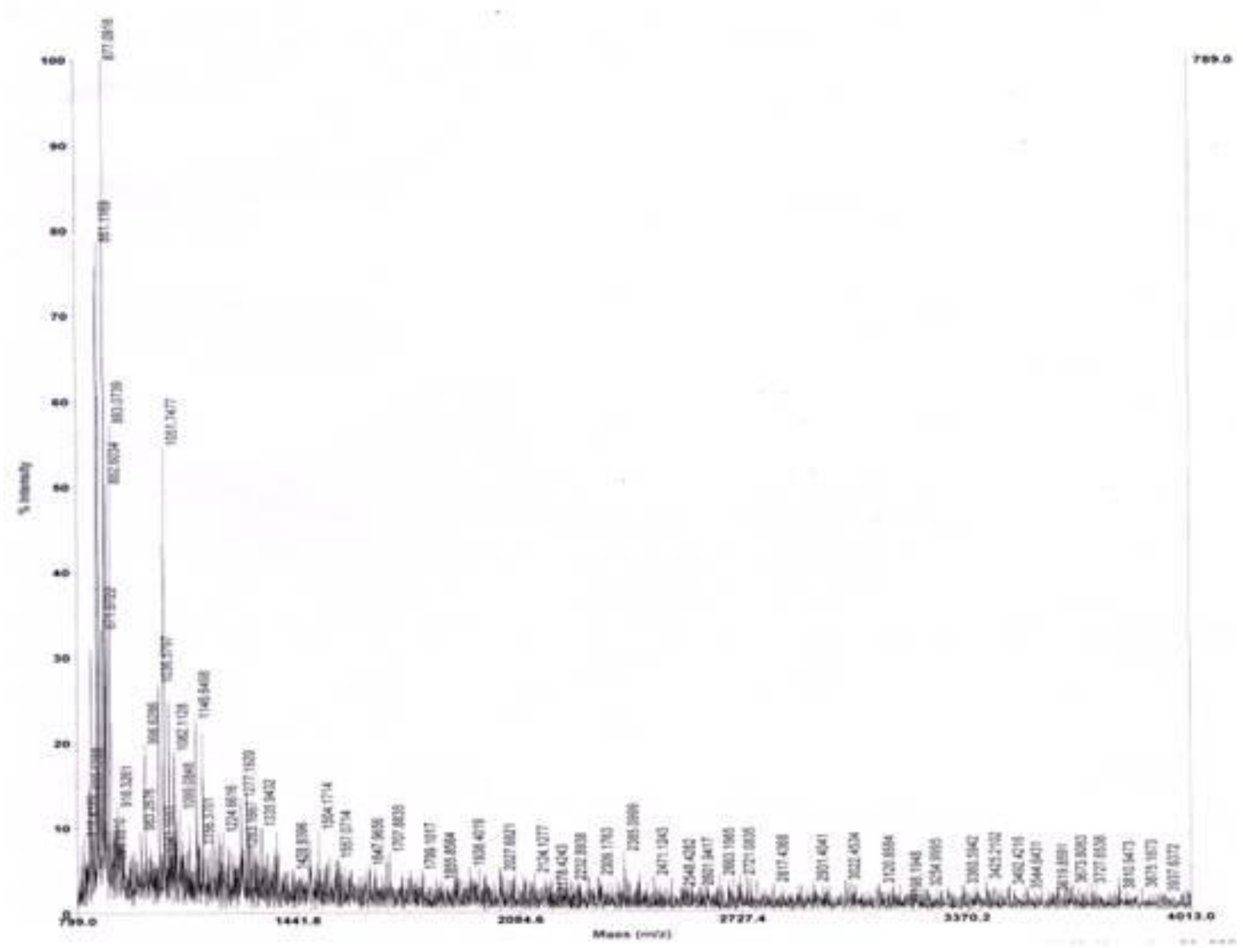

Figure S1. MALDI-TOF spectra of the tryptic fragments obtained from the peptide 34 kDa.

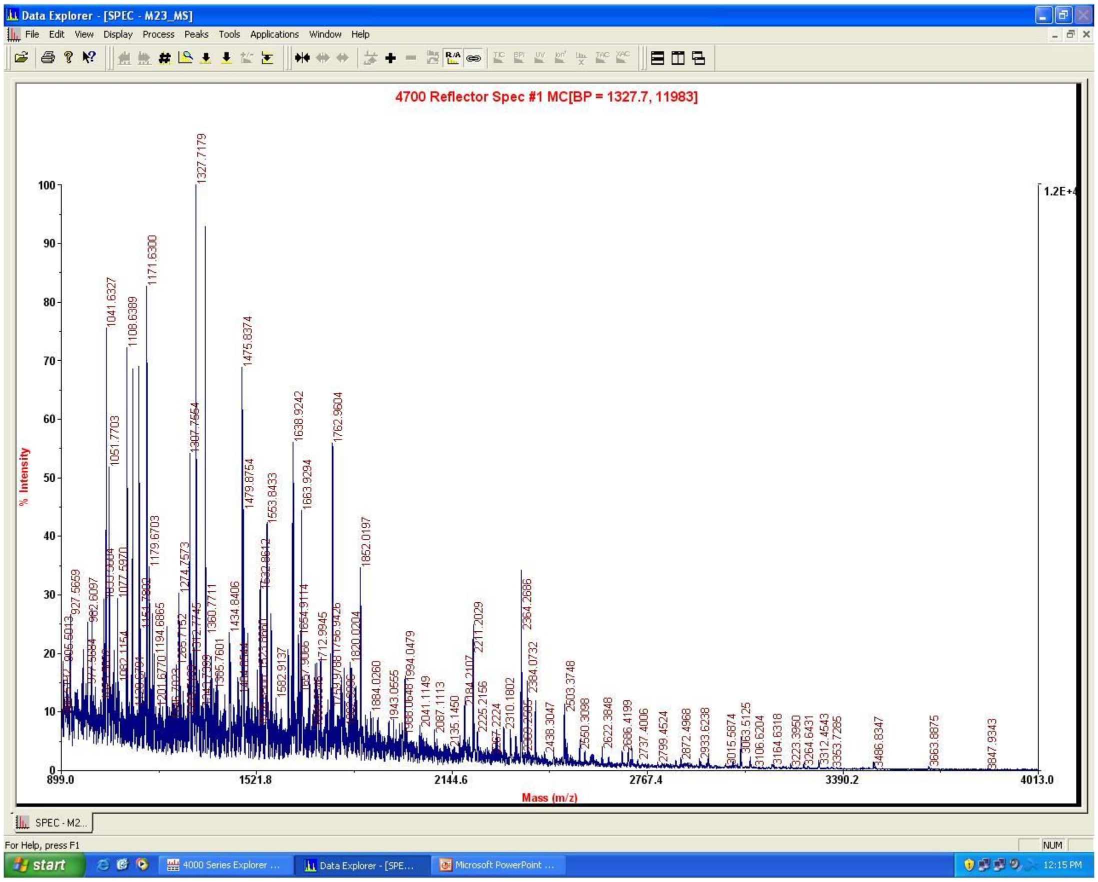

Figure S2. MALDI-TOF spectra of the tryptic fragments obtained from the peptide 45 kDa.

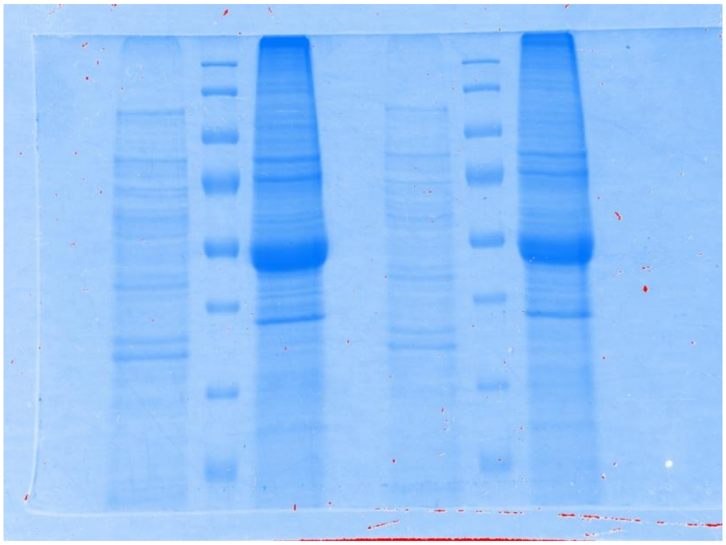

Original SDS-PAGE of gel for Figure 1

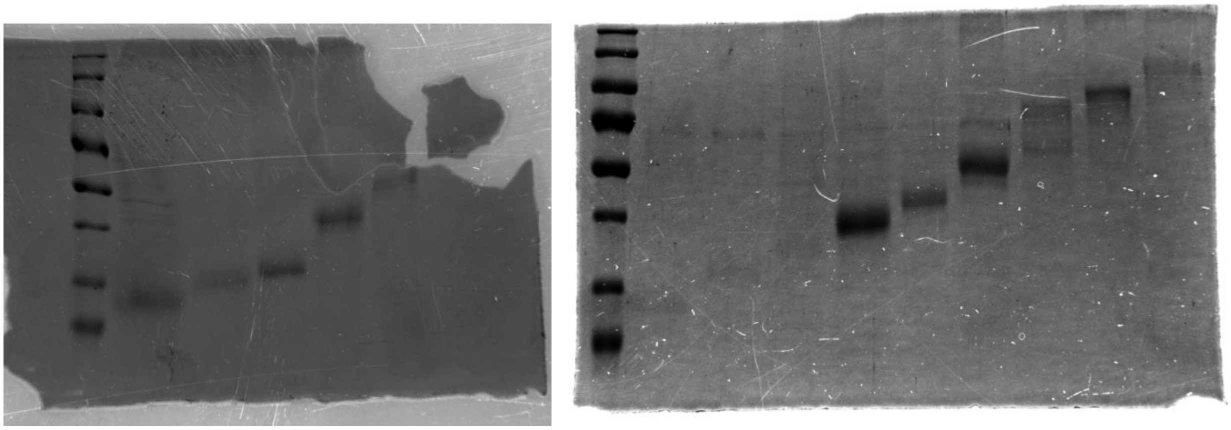

Original SDS-PAGE of gels for Figure 3
